# Effects of Sanguis Draconis on Perforator Flap Survival in Rats

**DOI:** 10.3390/molecules21101262

**Published:** 2016-09-26

**Authors:** Yang Zhang, Xiaobing Cai, Lifeng Shen, Xiaowen Huang, Xuping Wang, Yinan Lan, Dan Shou

**Affiliations:** 1 Department of Medicine, Zhejiang Academy of Traditional Chinese Medicine, Hangzhou 310007, Zhejiang, China; zhangyang0310@163.com (Y.Z.); bowen8883@126.com (X.H.); wangxp@hz.cn (X.W.); 2 Department of Orthopedic Surgery, Lishui Central Hospital, Lishui 323000, Zhejiang, China; caixiaobin1974@163.com (X.C.); nan295785528@126.com (Y.L.); 3 Department of Orthopedic Surgery, Zhejiang Provincial Tongde Hospital, Hangzhou 310012, Zhejiang, China; hzshenlf@163.com

**Keywords:** sanguis draconis, dracorhodin, perforator flaps, flap survival, perfusion, microvessels

## Abstract

Sanguis draconis, a resin known to improve blood circulation, relieve pain, stimulate tissue regeneration, and heal wounds, is widely used in clinical practice. In this study, we prepared an ethanol extract of sanguis draconis (EESD) containing 75.08 mg/g of dracorhodin. The experiment was carried out on 20 rats that were divided into two groups, a control group (*n* = 10) and an EESD group (*n* = 10). All the rats underwent a perforator flap surgery, after which post-operative abdominal compressions of EESD were given to the EESD group for seven days, while the control group received saline. Flap survival percentages were determined after seven days, and were found to be significantly higher in the EESD group than in the control group. Results of laser Doppler flowmetry (LDF) showed that perforator flaps in the EESD group had higher perfusion values than those of the control group. The flap tissues were stained with hematoxylin and eosin, followed by immunohistochemical evaluation. Superoxide dismutase (SOD) expression and micro-vessel development markedly increased in the EESD group, while malondialdehyde (MDA) levels decreased. This is the first study to investigate the effect of sanguis draconis on perforator flap survival. Our results demonstrate that sanguis draconis can improve perforator flap survival in rats by promoting microvessel regeneration and blood perfusion.

## 1. Introduction

Perforator flaps were first described by Koshima in 1989, and began to be widely used for the reconstruction of soft tissue defects when plastic surgeons recognized their usefulness as freestyle island flaps [1,2]. Various perforator flaps with versatile designs have been developed to replace the traditional flaps and reduce donor morbidity [3,4]. However, these “designed” perforator flaps are known for their short survival and deficient blood perfusion [5]. Enhancing the perfusion of perforator flaps can greatly improve the outcome of perforator flap surgeries, especially when there is a solitary perforator [6].

Sanguis draconis, a resin obtained from *Daemonorops draco* BL. (family Palmae), has long been used in China [7]. Pharmacological studies have shown that it could positively influence the treatment of blood stasis syndrome, trauma, tumors, inflammation, gynecopathy, allergic dermatitis, and other diseases [8]. In addition, reports have indicated that sanguis draconis has vasodilator, anti-inflammatory, and antioxidant activities in vivo and in vitro [9,10]. Previous studies have also reported that sanguis draconis could enhance the synthesis of transforming growth factor 1 (TGF-β1) and vascular endothelial growth factor (VEGF) [11,12]. Dracorhodin, the main component in sanguis draconis, was reported to induce vasodilatation. All these studies indicate that this resin has multiple pharmacological benefits that require further investigation. However, to the best of our knowledge, no studies have investigated its effects on perforator flaps, especially, the effects on the blood perfusion of remote flaps.

In this study, we tested the hypothesis that an ethanol extract of sanguis draconis (EESD) could improve the viability of perforator-based skin flaps in rats through its vasoregulatory effects. In addition, we investigate the effect of the administration route on the efficacy.

## 2. Results and Discussion

### 2.1. Quality Control of EESD

**Figure 1:**
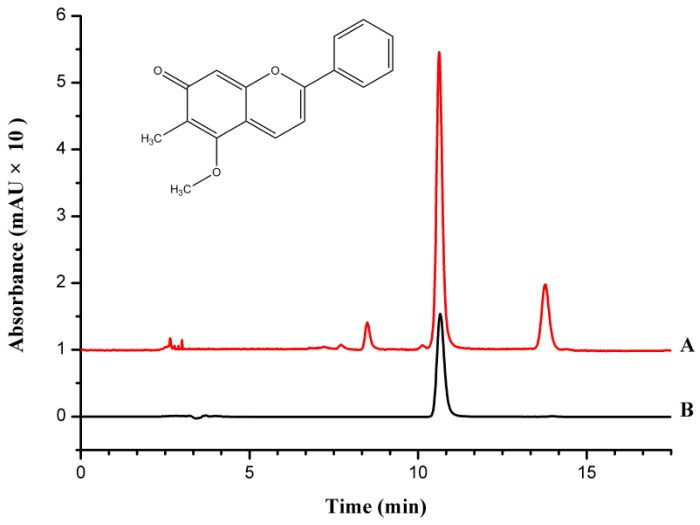
Chromatogram of dracorhodin in EESD (A) and in the standard (B).

The content of dracorhodin in sanguis draconis was stipulated by the Chinese pharmacopoeia as to be 10 mg/g; its content in EESD should not be less than 65.65 mg/g according to the extraction rate. Since dracorhodin perchlorate was used as the standard, the concentration of dracorhodin was calculated by dividing the concentration of dracorhodin perchlorate by 1.377. In this experiment, the content of dracorhodin in EESD was determined to be 75.08 mg/g. Chromatograms of the standard and EESD samples are shown in Figure 1.

### 2.2. Effect of EESD on Perforator Flap Survival

**Figure 2:**
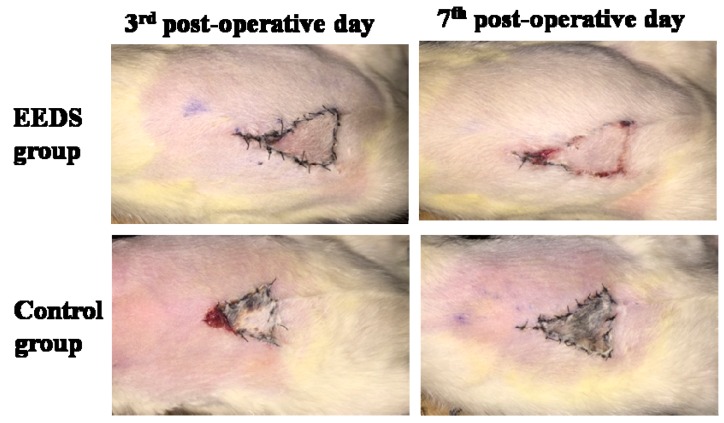
These digital images, taken on the third and seventh post-operative days, display the general form of perforator flaps in the EESD and control groups.

The general form of each flap was observed during the course of the treatment. On the first post-operative day, all flaps swelled to some extent, and the distal area C exhibited a dark purple color without obvious necrosis. On the third post-operative day, areas B and C in the control and EESD groups exhibited reddish brown focal or patchy necrosis with congestion. On the seventh post-operative day, the necrotic parts tended to fuse, scab, and harden. The boundaries between the necrotic and surviving parts were stable. Meanwhile, hair started to grow in the surviving portion, whereas the necrotic portion became hard, dark, glabrous, and did not bleed when cut with a scalpel (Figure 2). On the seventh post-operative day, the percentage of viable area (PVA) of EESD group was 98.02% ± 3.21%, significantly higher than that (5.36% ± 0.52%) of the control group (*p* < 0.01) (Table 1).

### 2.3. EESD’s Effect on Flap Perfusion

Flap necrosis is the ultimate indicator of flap ischemia and is directly correlated to inadequate blood supply. The mean perfusion values shown in Table 2 indicate that, on the seventh post-operative day, the perfusion in the EESD group was significantly higher than that in the control group. The perfusion values of area A in the EESD group were increased on the first and seventh post-operative day, while the perfusion values of area A in the control group were slightly decreased on the seventh post-operative day. The perfusion values of areas B and C in the control group markedly decreased on the first and seventh post-operative days.

### 2.4. EESD’s Effect on the Vascularization of the Perforator Flap

**Figure 3:**
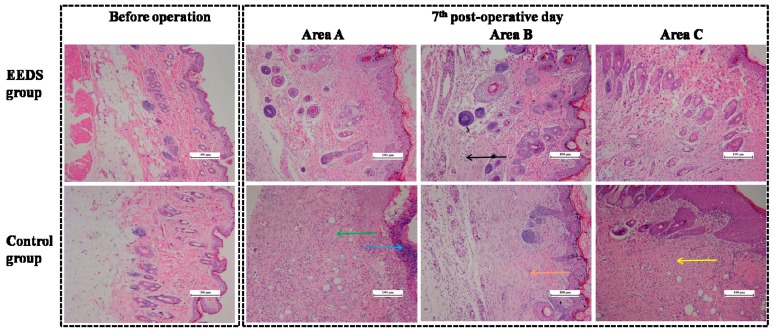
The hematoxylin and eosin stain images, taken before the operation and on the seventh post-operative day, display the histological changes in the middle areas A, B, and C of the flaps in the EESD and control groups. Magnification: × 100 (hematoxylin and eosin stain). The black arrow shows neovascularization, the blue arrow shows tissue necrosis, the green arrow shows tissue edema, the yellow arrow shows neutrophilic infiltration, and the orange arrow shows fibroblast proliferation.

**Figure 4:**
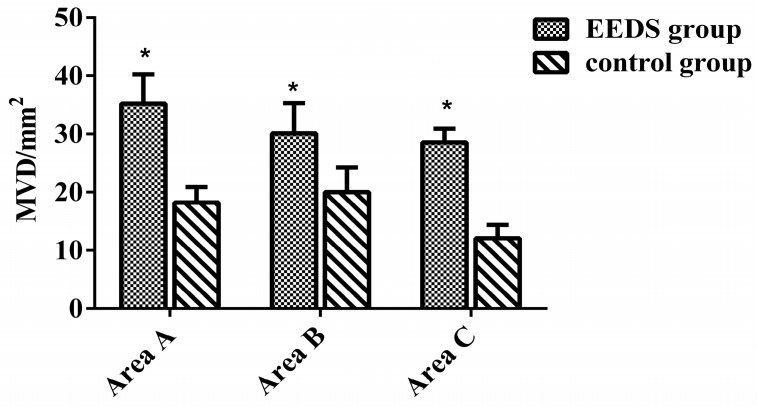
The histogram displays the MVDs (micro-vascular densities) of areas A, B, and C in the EESD and control groups. MVD, micro-vascular density. * *p* < 0.01 compared to the control group.

On the seventh post-operative day, histological examination of area C in the control group revealed full-thickness skin necrosis, structural damage, tissue edema, and fiber hyperplasia. Light microscopy detected a difference in areas B and C between the two groups. Inflammatory cell infiltration was detected in the control group but not in the EESD group, indicating that the inflammatory reaction in the treated group was less severe than that in the control group. For areas B and C, the EESD group exhibited higher proliferation of fibroblasts, thinner granulation tissue, more diffuse neutrophilic infiltration, and greater neovascularization than the control group. However, area A of the EESD group showed less marked tissue edema, vascular dilation, and inflammatory cell infiltration than the control group (Figure 3). As shown in Figure 4, the micro-vascular densities (MVDs) of area A were 35.22 ± 5.01 and 18.22 ± 2.69/mm^2^ in the EESD and control groups, respectively. The MVDs of area B were 30.12 ± 5.21 and 30.01 ± 4.21/mm^2^ in the EESD and control groups, respectively. The MVDs of area C were 28.56 ± 2.33 and 12.09 ± 2.30/mm^2^ in the EESD and control groups, respectively.

### 2.5. EESD’s Effect on the Expression of VEGF in the Skin Flap

**Figure 5:**
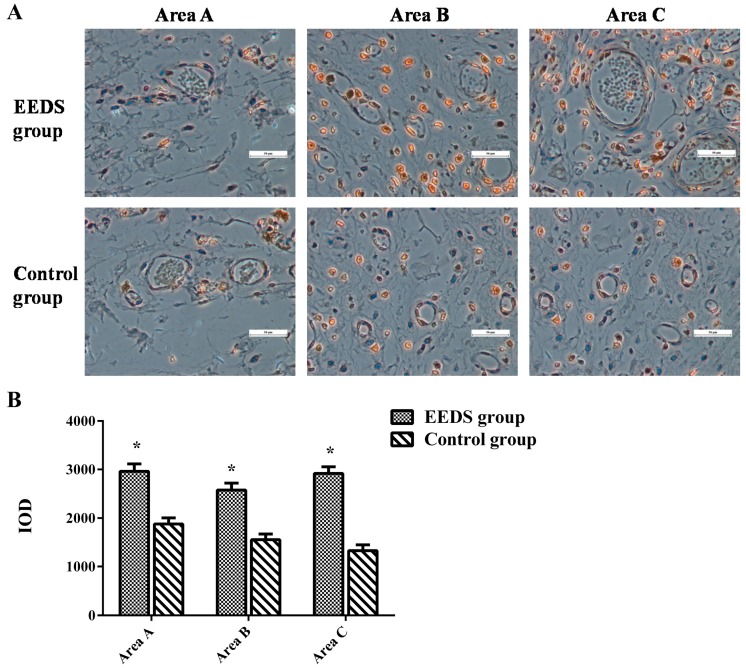
(**A**) The immunohistochemical stain images, taken before the operation and on the seventh post-operative day, display the expression of VEGF (vascular endothelial growth factor) in the perforator flaps; (**B**) The histogram displays the IOD (integrated optical density) valued of VEGF in the two groups. * *p* < 0.01 compared to the control group.

The integrated optical density (IOD) values that evaluate vascular endothelial growth factor (VEGF) expression in the EESD and control groups are shown in Figure 5A,B. The values indicate that VEGF expression in the EESD group was higher than in the control group (*p* < 0.01).

### 2.6. EESD’s Effect on Superoxide Dismutase and Malondialdehyde Levels

**Figure 6:**
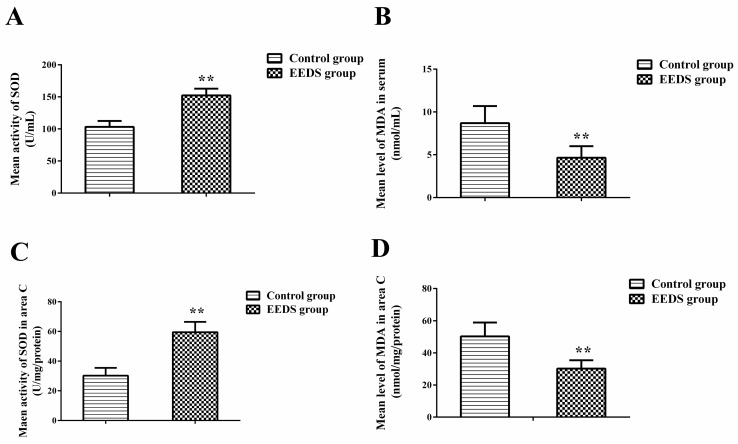
(**A**) The histogram displays the SOD (superoxide dismutase) serum activity in the EESD and control groups; (**B**) The histogram displays the MDA (Malondialdehyde) serum levels in the EESD and control groups; (**C**) The histogram displays the SOD activities in area C of the skin flap in the EESD and control groups; (**D**) The histogram displays the MDA levels in area C of the skin flap in the EESD and control groups. ** *p* < 0.01 compared to the control group.

The effect of EESD on superoxide dismutase (SOD) and malondialdehyde (MDA) levels is shown in Figure 6. EESD altered SOD and MDA levels, which suggests its protective effect against ischemia/reperfusion injury. The mean serum SOD activity in the EESD group was 152.32 ± 10.40 U/mL, which is significantly higher than that of the control group. However, the mean serum MDA level in the EESD group was 4.65 ± 1.36 nmol/mL, which is significantly lower than that of the control group. The mean SOD activity in area C of the skin flap in the EESD group was 59.47 ± 6.98 U/mg/protein, which is significantly higher than that of the control group. However, the mean level of MDA in area C of the skin flap in the EESD group was 30.21 ± 5.22 nmol/mg/protein, which is significantly lower than that of the control group.

### 2.7. Discussion

Perforator skin flaps are frequently used in plastic surgery to reconstruct structural defects, restore function, and improve skin appearance. However, ischemia and necrosis usually occur in distal flaps due to inadequate blood supply, leading to ischemia/reperfusion injury and venous congestion [13]. Furthermore, several medical methods have been investigated in previous studies; however, they failed to find an application for perforator flaps in routine practice.

In Chinese medicine, sanguis draconis is used as a “blood-activating panacea” that has great medicinal value. Its biological activity results mainly from its phenolic content. Dracorhodin is the principal bioactive component in sanguis draconis. However, it is a flavylium compound belonging to the anthocyanin family, which is unstable in solutions and unavailable as a standard substance. Dracorhodin perchlorate was chosen as the index component in the quality control of EESD. In addition, anthocyanins were proved to have antioxidant, anti-inflammatory, and angiogenic effects [14,15]. In this study, the distal flap areas in the control group became ischemic and necrotic on the third post-operative day, and the whole flaps became necrotic on the seventh post-operative day. The mean survival ratios of the perforator flaps showed that the EESD group had better survival than the control group. Results supporting the effects on ischemia/reperfusion injury at the cellular level have also been reported [16,17]. After the operation, the flaps in the EESD group appeared swollen, and their borders were contracted. This is an essential step in the process of wound healing, which is related to ischemia [18]. This suggests that, although the borders of the flaps were subjected to ischemia, they survived with the help of EESD.

Flap viability is directly correlated to adequate circulation [19]. Laser Doppler flowmetry is a reliable, accurate, and noninvasive method for continuous monitoring of the circulation (perfusion) in flaps [20]. Its results showed that the perfusion of the distal flap areas (B and C) of the EESD group was higher than that of the control group on the seventh post-operative day. These results indicate that EESD enhances the circulation capability of the epigastric artery perforator, the microvascular density of the distal perforator flap, and the microcirculation of the skin flap. The microcirculation improvement was caused by phlebarteriectasia and vascular remodeling. Perfusion values near the epigastric artery perforator (area A) in both the EESD and control groups on the first post-operative day were significantly higher than before the operation. This might be attributed to acute ischemia in the flaps, which results in a brief opening of the collateral vessels and emergence of new blood vessels to compensate for this ischemia.

Angiogenesis plays a crucial role in the matrix formation during the flaps’ healing process. It involves development of endothelial cells including keratinocytes, macrophages, and fibroblasts from parent blood vessels, followed by migration, proliferation, and anastomosis to other vessels [21]. VEGF has been reported to play an important role in the production and development of these endothelial cells, and it is the fundamental mediator of both neovascularization and angiogenesis [22,23]. When VEGFR1 and VEGFR2 receptors present on the endothelial cells are activated by binding to VEGF, angiogenesis is induced [24]. Previous reports have shown that administration of VEGF injections at random in skin flaps improved their survival rates, which suggests that there is a strong correlation between high and consistent VEGF expression and better flap survival after ischemia/reperfusion injury [25]. In our study, we confirmed the higher integral absorbance (IA) value in the EESD group than in the control group by immunohistochemical staining. Our results indicated that the higher survival ratio in the EESD group is likely related to angiogenesis activated by VEGF, which protects against ischemia/reperfusion injury. However, for a better understanding of the mechanisms of action of EESD in wound healing, future investigation should focus on the VEGF-related pathways, such as the MAPK, PI-3K-AKT/PKB, and NO signaling pathways [26,27], as well as the signaling molecules mediating EESD activity in the rat random skin flap model. The MVD, a marker of angiogenesis, was higher in the EESD group than in the control group. Similarly, flap angiograms showed that EESD-treated flaps contained a greater number of micro vessels, indicating that EESD might improve flap survival via VEGF-mediated angiogenesis.

Ischemia and necrosis involve apoptosis, oxygen-free radicals, platelet aggregation, and leukocyte-endothelium interactions; they result from acute interruption of blood flow within the microvasculature [28]. The SOD activities and MDA levels in the EESD group were significantly different from those in the control group. SOD is an important metalloprotein antioxidant enzyme that removes O^2−^ (the predecessor of H_2_O_2_ and OH^−^), thus protecting cells from injury by toxic oxygen-derived free radicals [29]. Functionally, SOD as a generator of extracellular H_2_O_2_ stimulates endothelial cell migration and proliferation and promotes angiogenesis by promoting VEGFR2 signaling [30]. Generally, free radicals lead to the peroxidation of lipids and protein, and damage cells and organelle membranes, thereby compromising tissue structure and function [31]. MDA is a product of lipid peroxidation and is therefore a marker of tissue injury [32]. The generation of oxygen-derived free radicals is one of the most important pathogenic mechanisms of ischemia/reperfusion injury. Various studies have demonstrated that a burst of oxygen-free radicals attacks plasma membrane lipids and proteins within the first few minutes of reperfusion. In addition, reperfusion leads to the accumulation of activated neutrophils in ischemic tissue, and activation of xanthine oxidase in endothelial cells, resulting in a rapid flap necrosis. We confirmed that EESD has protective effects against endogenous SOD activity and thus inhibits the effect of lipid peroxidation. Furthermore, sanguis draconis resists ischemic/reperfusion injury by its free radical scavenging effects.

## 3. Experimental Section

### 3.1. Materials and Chemicals

Crude sanguis draconis was purchased from Huadong Medicine Co. Ltd. (Hangzhou, Zhejiang, China). All chemicals used, including solvents, were of analytical grade. Dracorhodin perchlorate was acquired from the National Institutes for Food and Drug Control (Lot Number: 110811-201506, Beijing, China). Superoxide dismutase (SOD) and malondialdehyde (MDA) ELISA kits were purchased from Beyotime Biotechnology Co. Ltd. (Shanghai, China). Anti-VEGF polyclonal antibodies were obtained from Proteintech Group, Inc. (Wuhan, China). Goat anti-rabbit immunoglobulin G (Santa Cruz Biotechnology, Santa Cruz, CA, USA) was obtained as a secondary antibody.

### 3.2. Animals and Groups

Healthy male Sprague-Dawley rats (250 ± 20 g) were purchased from the Experimental Animal Center of Zhejiang Province (license no. SCXK 2014-0003). All the animal experiments were performed in strict accordance with the Guide for the Care and Use of Laboratory Animals and were approved by the Bioethics Committee of Zhejiang Academy of Traditional Chinese Medicine. Rats were divided randomly into EESD group and control group, 10 rats per group. After operation and observation, all rats were euthanized with an overdose of pentobarbital sodium.

### 3.3. Preparation of Ethanol Extract of Sanguis Draconis (EESD)

Sanguis draconis power (30 g) was extracted twice with 95% ethanol (at a ratio of 1:5, *w*/*v*) for 30 min at room temperature, with the assistance of ultrasound, and then filtered. The extracted solutions were combined and concentrated to 1.0 g/mL crude drug. Normally, 4.57 g of EESD can be obtained from 30 g of sanguis draconis. Dracorhodin in EESD was quantitatively analyzed by high-performance liquid chromatography (HPLC) in a previously reported research [12]. Dracorhodin perchlorate was purchased as a standard. EESD was stored at 4 °C for future administration to rats.

### 3.4. Perforator Flap Animal Model and EESD Administration

**Figure 7:**
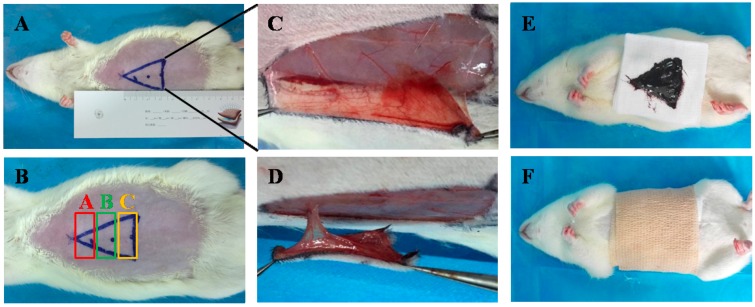
(**A**) The triangular boundary on the abdominal skin; (**B**) the perforator flaps divided into three zones: top (area A), intermediate (area B), and distal (area C) zones; (**C**) the epigastric artery on the perforator flap; (**D**) the epigastric perforator flap with a solitary perforator; (**E**,**F**) EESD applied on the perforator flap after the operation.

Rats were anesthetized by an intraperitioneal injection of 2% (*w*/*v*) pentobarbital sodium (Merck. Co., Darmstadt, Germany) at a dose of 40 mg/kg. A model of modified perforator flap was established in the rat abdomens (at the same position in all rats) [33]. We outlined a triangle on the abdominal skin of each rat, which was then cut (Figure 7A). The vertex of the triangle was 1 cm under the xiphoid process, its lower border connected the two anterior superior iliac spines, and its lateral sides joined the preceding points. First, flaps were cut from the right side skin to the right side of the rectus muscle surface. There were four epigastric artery perforators arranged vertically (Figure 7C). The second source’s cranial epigastric vessels and intercostal perforating vessels were retained, and the others were cut off. Therefore, the flap was supplied by blood from one epigastric artery perforator. The skin flap was sutured in situ by 3/0 sutures. The perforator flaps were divided into three zones (Figure 7B): top (area A), intermediate (area B), and distal (area C) zones. Following the surgical procedure, a 3 cm^2^ fabric evenly coated with 0.62 g EESD was attached to the wound of each rat and was changed every day (0.21 g/cm^2^/d) for seven successive days(Figure 7E,F). Simultaneously, the rats of the control group received saline.

All rats were individually housed after surgery to prevent injury from cannibalism or normal socialization activities. Each rat was fitted with a neck collar to prevent self-mutilation. These measures were undertaken by a blinded researcher. No rats died during the procedure.

### 3.5. General Observation and Flap Assessment

Flap survival was observed during the seven days, and macroscopic changes including appearance, color, texture, and hair condition were noted. On the third and seventh post-operative days, the surviving flap areas were measured by imaging the flaps and superimposing the images on graph paper. All results are expressed as percentages of viable area (PVA) calculated from the following equation:

(1) $$\text{PVA}=\frac{\text{Av}}{\text{At}}\times 100\%$$

where Av is the viable area and At is the total area (viable and ischemic).

### 3.6. Measurement of Perfusion Values with Laser Doppler Flowmetry

On the first and seventh post-operative days, the rats were anesthetized by an intraperitoneal injection of 2% (*w*/*v*) pentobarbital sodium (40 mg/kg). The perfusion values of areas A, B, and C of the perforator flaps were determined by laser Doppler flowmetry (LDF) [34] by selecting three measuring points in the three areas. A laser probe was affixed by an adhesive tape to each selected measuring point and in turn connected to a model LDF for constant flow monitoring. The perfusion values of each rat were also recorded before the operation.

### 3.7. Hematoxylin and Eosin Staining

After seven days, all animals were euthanized with an overdose of chloral hydrate, and the flap tissues were dissected. The tissue samples from each areas (A, B and C) from each group were collected, post-fixed in 4% (*v*/*v*) paraformaldehyde for 24 h, and embedded in paraffin wax for transverse sectioning. Sections (4 µm in thickness) were prepared for hematoxylin and eosin staining. Granulation tissue thickness, tissue edema, and neutrophil infiltration were observed under a light microscope (100 and 200 magnification), and the number of microvessels per unit area (mm^2^) was calculated as an indicator of MVD. Transmitted light images of the stained sections were taken using a microscope (Olympus-CX41, Olympus, Tokyo, Japan) connected to a CCD camera (DP72; Olympus), and the images were recorded using cellSens standard software (Olympus).

### 3.8. Immunohistochemistry

Five section specimens of areas A, B, and C from each group were deparaffinized in xylene and rehydrated through a graded set of ethanol baths. After washing, the sections were blocked with 3% (*v*/*v*) H_2_O_2_ and treated with 10.2 mM sodium citrate buffer (antigen retrieval) for 20 min at 95 °C. After being blocked with 5% (*w*/*v*) bovine serum albumin and 1% (*v*/*v*) tween-20 in phosphate-buffered saline for 10 min, the sections were incubated with antibodies against vascular endothelial growth factor (VEGF), overnight at 4 °C. Finally, the sections were incubated with an appropriate HRP-conjugated secondary antibody and counterstained with hematoxylin. Flap tissues were imaged at × 400 magnification using an auto-analysis imaging system (Olympus-CX41, Olympus). The data were quantified using a medical image management system (Image-Pro Plus, IPP6.0).

### 3.9. Superoxide Dismutase Activity and Malondialdehyde Content

On the seventh post-operative day, five rats from each group were anesthetized with 2% (*w*/*v*) pentobarbital sodium, and blood was drawn from the tail vein. Tissue samples (0.5 cm × 0.5 cm) from area C of both groups were weighed, homogenized, and diluted to 10% (*v*/*v*) in an ice bath. SOD activity was measured using the oxidase enzymatic method and the MDA content was determined according to the spectrophotometric presence of thiobarbituric acid reactive substances [35].

### 3.10. Statistical Analysis

All results are expressed as means ± standard deviation. All data were analyzed using the Statistical Package for the Social Sciences (SPSS) for Windows software package (ver. 19.0 SPSS Inc., Chicago, IL, USA). Graphs were rendered using the Graph Pad Prism software package (ver. 5.0, Graph Pad Software, Inc., La Jolla, CA, USA). A *p*-value < 0.05 was considered to indicate statistical significance. The degree of necrotic change and the histological results were compared between the two groups using the Mann–Whitney test.

## 4. Conclusions

Our results indicate that the administration of EESD on the perforator flap improves its viability. The decreased capillary volume has been attributed to the anti-neoangiogenic effects of EESD. In conclusion, we believe that local transdermal administration of sanguis draconis can enhance flap survival.

## Figures and Tables

**Table 1:** The mean proportion of survival area after operation (*n* = 5).

Group	Proportion of Survival Area (%)	
	3rd Post-Operative Day	7th Post-Operative Day
EESD group	82.32 ± 3.07 *	98.02 ± 3.21
Control group	60.05 ± 2 ^#^	5.36 ± 0.52

Data are presented as mean ± SD. * *p* < 0.05 compared to the control group; ^#^ *p* < 0.01 compared to the control group.

**Table 2:** The mean perfusion values (PU) measured by LDP after operation (*n* = 5).

Time	EESD Group			Control Group		
	Area A	Area B	Area C	Area A	Area B	Area C
Before operation	46.25 ± 2.14	50.23 ± 3.01	48.12 ± 2.86	48.32 ± 2.46	51.36 ± 3.69	49.65 ± 3.47
1 day after operation	59.61 ± 2.04 ^※^	48.69 ± 1.38 *	51.27 ± 3.46 *^,#^	56.33 ± 2.91 ^※^	33.26 ± 2.52 ^▲^	22.64 ± 2.01 ^▲^
7 day after operation	65.86 ± 1.47 *^,#,※^	60.85 ± 4.61 *^,#,※^	58.41 ± 4.07 *^,#,※^	43.95 ± 3.81	10.67 ± 1.56 ^▲^	10.57 ± 1.40 ^▲^

Data are presented as mean ± SD. * *p* < 0.05 compared to the control group, ^#^ *p* < 0.01 compared to the control group, ^※^ *p* < 0.05 compared to before operation, ^▲^ *p* < 0.01 compared to before operation.
